# TCCR/WSX-1 is a novel angiogenic factor in age-related macular degeneration

**Published:** 2012-01-28

**Authors:** Ho Jin Sung, Jung Il Han, Ji Won Lee, Ki Bang Uhm, Kyun Heo

**Affiliations:** 1 Functional Genomics Branch, Division of Convergence Technology, National Cancer Center, Gyeonggi-do, Republic of Korea; 2 Department of Life Science, Division of Life and Pharmaceutical Sciences, and Center for Cell Signaling and Drug Discovery Research, Ewha Womans University, Seoul, Republic of Korea; 3 Department of Ophthalmology, Retina Center, Kim’s Eye Hospital, Konyang University School of Medicine, Seoul, Republic of Korea; 4 Department of Ophthalmology, Hanyang University College of Medicine, Seoul, Republic of Korea

## Abstract

**Purpose:**

Age-related macular degeneration (AMD) is the major cause of blindness among persons aged 60 years and older. The current approved therapies for AMD are exclusively limited to inhibiting vascular endothelial growth factor. However, substantial improvement in vision occurs in only one-third of patients treated with vascular endothelial growth factor antagonists, and one-sixth of treated patients still progress to legal blindness. Therefore, more specific targets are needed to treat AMD. Our goal was to find secretory proteins that change in number in the aqueous humor and that cause exudative AMD disease.

**Methods:**

The number of molecules changed in the aqueous humor of patients with AMD compared to the control group was determined using antibody array analysis. The levels of angiopoietin-2 and insulin-like growth factor binding protein-related protein 7 were measured using enzyme-linked immunosorbent assay. The levels of T-cell cytokine receptor (TCCR/WSX-1) were determined using western blot. Potential TCCR/WSX-1-mediated effects on tube formation as well as phosphorylation of extracellular signal-regulated kinase in human umbilical vein endothelial cells were determined.

**Results:**

We found that the numbers of several molecules were changed in the aqueous humor of patients with AMD compared to the control group. Among them, angiopoietin-2 was reduced by 20% and TCCR/WSX-1 was increased twofold. Moreover, exogenous TCCR protein induced tube formation and phosphorylation of extracellular signal-regulated kinase in human umbilical vein endothelial cells.

**Conclusions:**

Our study suggests that TCCR/WSX-1 is closely associated with angiogenesis and could serve as a novel therapeutic target in patients with AMD.

## Introduction

Exudative age-related macular degeneration (AMD) with characteristic choroidal neovascularization (CNV) causes irreversible loss in visual acuity in elderly patients [1,2]. Treatments currently available for the disease include laser photocoagulation, verteporfin photodynamic therapy, and intravitreal injections of corticosteroids and antiangiogenic agents [3,4]. Many studies have reported the benefits of each treatment, although none is without risks. Because AMD is complex, the cause of the disease is largely unknown.

Currently, numerous studies have demonstrated that the concentrations of various secretory molecules are increased and caused the disease in patients with AMD: Interleukin (IL)-6, IL-8, IL-10, C-reactive protein, monocyte chemotactic protein (MCP)-1, and vascular endothelial growth factor A (VEGF-A) [5-8]. Among these molecules, proangiogenic factor VEGF-A has been validated in patients with AMD with CNV, and VEGF-A-neutralizing agents such as bevacizumab (Avastin), ranibizumab (Lucentis), and pegaptanib (Macugen) have recently been studied for treating the disease [9,10]. However, problems remain with its curative value and safety concerns, and targets for CNV that are more specific are needed.

Thus, the purpose of the present study is to investigate changes in the concentration of various secretory molecules in the aqueous humor of patients with AMD and to determine whether these molecules could induce angiogenesis.

## Methods

### Preparation of patient samples

This clinical study investigated the levels of various proteins in the aqueous humor of patients with CNV secondary to AMD and in normal control cataract patients. This study was approved by the Institutional Review Board of Kim’s Eye Hospital, Seoul, Korea. Informed consent was obtained from all participants. Patients were enrolled from the Retina Center at Kim’s Eye Hospital from March 2010 to August 2010. Patients (male: 22, female: 13) over 50 years old with active CNV secondary to AMD identified with fluorescein angiogram who underwent injections of 0.5 mg of ranibizumab with paracentesis of the aqueous humor before injection were included in this study. Exclusion criteria were as follows: (1) ocular disease apart from AMD and cataract; (2) previous ocular surgery apart from cataract surgery, photodynamic therapy with verteporfin, and intravitreal triamcinolone injection; and (3) cataract surgery, intravitreal triamcinolone injection, and photodynamic therapy within 6 months before entry in the study. Undiluted aqueous humor samples (100–150 μl) were obtained through anterior chamber paracentesis from 30 eyes in 35 patients with AMD. All patients did not have significant systemic diseases. Undiluted aqueous humor (100–150 μl) was also obtained as a control sample from 24 eyes immediately before cataract surgery. Samples were stored at −80 °C until analysis. No significant statistical difference was noted in the mean age of the age-related macular degeneration group (67.9±8.7 years) versus the control group (64.4±10.7 years).

### Membrane-based human antibody array

In this study, we examined the differences in protein expression levels between the aqueous humor of controls and patients with AMD by profiling the proteins using RayBio^®^ Biotin Label-based Human Antibody Array I (Catalog No: AAA-BLM-1–2; Ray Biotech, Inc., Norcross, GA). The array membrane is spotted with 507 specific antibodies toward cytokines, chemokines, adipokines, growth factors, angiogenic factors, proteases, soluble receptors, soluble adhesion molecules, and other proteins in culture supernatant and serum. Biotinylated proteins of samples can be captured by these specific antibodies and horseradish peroxidase (HRP)-conjugated streptavidin in a sandwich format. After enhanced chemiluminescence (ECL) solution was added, the signals can be visualized with chemiluminescence. The assay for antibody arrays was performed according to the manufacturer’s instructions. Briefly, to biotinylate the samples, 50 μl of each undiluted aqueous humor sample (control or patients with AMD, 24 cases each) was mixed and dialyzed with phosphate buffer saline (PBS; 137 mM NaCl, 2.7 mM KCl, 10 mM Na_2_HPO_4_, 2 mM KH_2_PO_4_, pH 7.4), and an internal control was added to the dialyzed samples. After 1.5 μl of labeling reagent was added to the samples and they were incubated at room temperature (RT) for 30 min, the reaction was stopped with 5 μl of stop solution. Free biotin was removed with the spin column, and the biotin-labeled samples were diluted with 7 ml of blocking buffer. Array membranes were blocked with 8 ml of 1× blocking buffer at RT for 1 h, and then incubated with the biotin-labeled samples at 4 °C overnight. Then the membranes were washed three times with 20 ml of wash buffer I and II, followed by incubation with 8 ml of 500-fold diluted HRP-conjugated streptavidin for 2 h at RT. After the membranes were washed, mixed detection buffer was loaded onto the membranes to cover the entire surface, and they were incubated for 2 min. Finally, spot signals were visualized by exposing the membranes to radiographic film (Kodak X-Omat^TM^; Kodak, Rochester, NY) for variable times. To select the target molecules showing significantly different spot signal intensities between controls and patients with AMD, results from multiple films were compared by three persons in a blinded manner. Biotinylated and non-biotinylated proteins placed on the membranes were used as positive and negative controls, and internal controls were used to monitor the whole process including biotin-labeling.

### Protein quantitation

The levels of angiopoietin-2 and insulin-like growth factor binding protein 7 (IGFBP-7) were measured using sandwich enzyme-linked immunosorbent assay (ELISA) according to the respective manufacturers’ protocols. To measure the level of angiopoietin-2, 30 μl of undiluted aqueous humor of controls or patients with AMD (18 cases for control and 13 cases for patients with AMD) were prediluted with 70 μl of standard diluent buffer supplied with the Human Angiopoietin-2 ELISA kit (Invitrogen, Camarillo, CA). One hundred μl of prediluted samples were added to each well filled with 25 μl of incubation buffer and then incubated with 50 μl of biotinylated anti-Ang-2 solution at RT for 2 h. After the wells were aspirated and washed four times, 100 μl of streptavidin-HRP working solution was added to each well and incubated at RT for 30 min. Then the wells were washed four times and incubated with 100 μl of stabilized chromogen at RT for 30 min in the dark. The reaction was stopped with 100 μl of stop solution, and then the absorbance was read at 450 nm. To assay the level of IGFBP-7, the Human IGF-BP7 “SUPER X” ELISA kit was purchased from Antigenix America (Huntington, NY). In brief, 5 μl of each undiluted aqueous humor sample (30 cases for control and 23 cases for patients with AMD) was diluted with 95 μl of PBS containing 0.05% Tween-20 and 0.1% BSA, loaded into each precoated well, and incubated at RT for 2 h. After the wells were aspirated and washed four times, 100 μl of 0.1 μg/ml of biotin-labeled tracer antibody was added and incubated at RT for 1 h. After the solution was decanted, each well was washed four times, 100 μl of streptavidin-HRP conjugate (1:2,000 in diluent) was added, and the well was incubated at RT for 30 min. TMB substrate solutions A and B were mixed (1:1) just before being added and loaded into each well, which was washed four times and then incubated at RT for 10 min. Color development was stopped by adding 100 μl of stop solution, and the absorbance was read at 450 nm.

The TCCR level was measured with western blot. Briefly, 15 μl of undiluted aqueous humor of controls or patients with AMD was heated in sodium dodecyl sulfate (SDS) sample buffer (Laemmli) for 5 min at 94 °C, and subjected to SDS–PAGE based on the Tris-Aspartate buffer system and then transferred to nitrocellulose membrane. To minimize non-specific binding, the membrane was blocked for 1 h at RT with 5% (w/v) skimmed milk in TBS-T buffer (25 mM Tris-HCl, 150 mM NaCl [pH 7.5], and 0.05% Tween-20) and then incubated overnight at 4 °C with primary antibody to TCCR (1:200; R&D Systems; Minneapolis, MN) in TBS-T containing 5% skimmed milk. Then the membrane was washed in TBS-T and probed for 1 h at RT with horseradish peroxidase-conjugated antigoat secondary antibody (1:5,000; Vector Laboratories; Burlingame, CA) in TBS-T containing 5% skimmed milk. After being washed in TBS-T, the immunoreactive bands were visualized with ECL detection reagent (Pierce; Rockford, IL).

To assay the expression levels and phosphorylation status of p44/42 MAPK and β-actin, HUVECs were cultured in 12-well plates coated with 1% gelatin and then treated with 100 pM recombinant human TCCR/WSX-1/Fc chimera protein or human immunoglobulin G (IgG) Fc protein for 2, 5, or 30 min. After 100 μl of SDS sample buffer was added, the samples were subjected to western blot using antiphospho-p44/42 MAPK (p-ERK, 1:1,000; Cell Signaling Technology; Danvers, MA), p44/42 MAPK (ERK, 1:2,000; Cell signaling), or β-actin (1:1,000; Santa Cruz Biotechnology; Santa Cruz, CA) antibodies.

### Cell culture

HUVECs were obtained from ATCC (Manassas, VA) and grown (37 °C, 5% CO_2_) in M199 (Thermo Fisher Scientific; Waltham, MA) with 20% fetal bovine serum (Thermo Fisher Scientific) and 3 ng/ml basic fibroblast growth factor (bFGF; Sigma-Aldrich; St. Louis, MO). The cells were studied between passages 4 and 7.

### Tube formation assay

Tube formation was performed by placing ice-cold growth factor-reduced Matrigel matrix (8.2 mg/ml; BD Biosciences; Franklin Lakes, NJ) in a prechilled 96-well plate (50 μl/well) and incubating it at 37 °C for 30 min to allow polymerization. Thereafter, HUVECs (1×10^4^ cells/well) were seeded on a Matrigel-coated 96-well plate and incubated in the M199 media for 8 h in the presence or absence of 20% fetal bovine serum, different concentrations (10, 100, 1,000 pM) of recombinant human TCCR/WSX-1/Fc chimera protein (R&D Systems), or human IgG Fc protein (R&D Systems). Tube-like structures were observed after 8 h and photographed under phase contrast microscopy using an inverted Olympus microscope (Tokyo, Japan) at 50× magnification. Quantification of the images was performed in a blinded manner by counting total tubes per five fields. The experiment was performed three times in triplicate wells.

## Results

### Analysis of secretory proteins in aqueous humor

**Figure 1:**
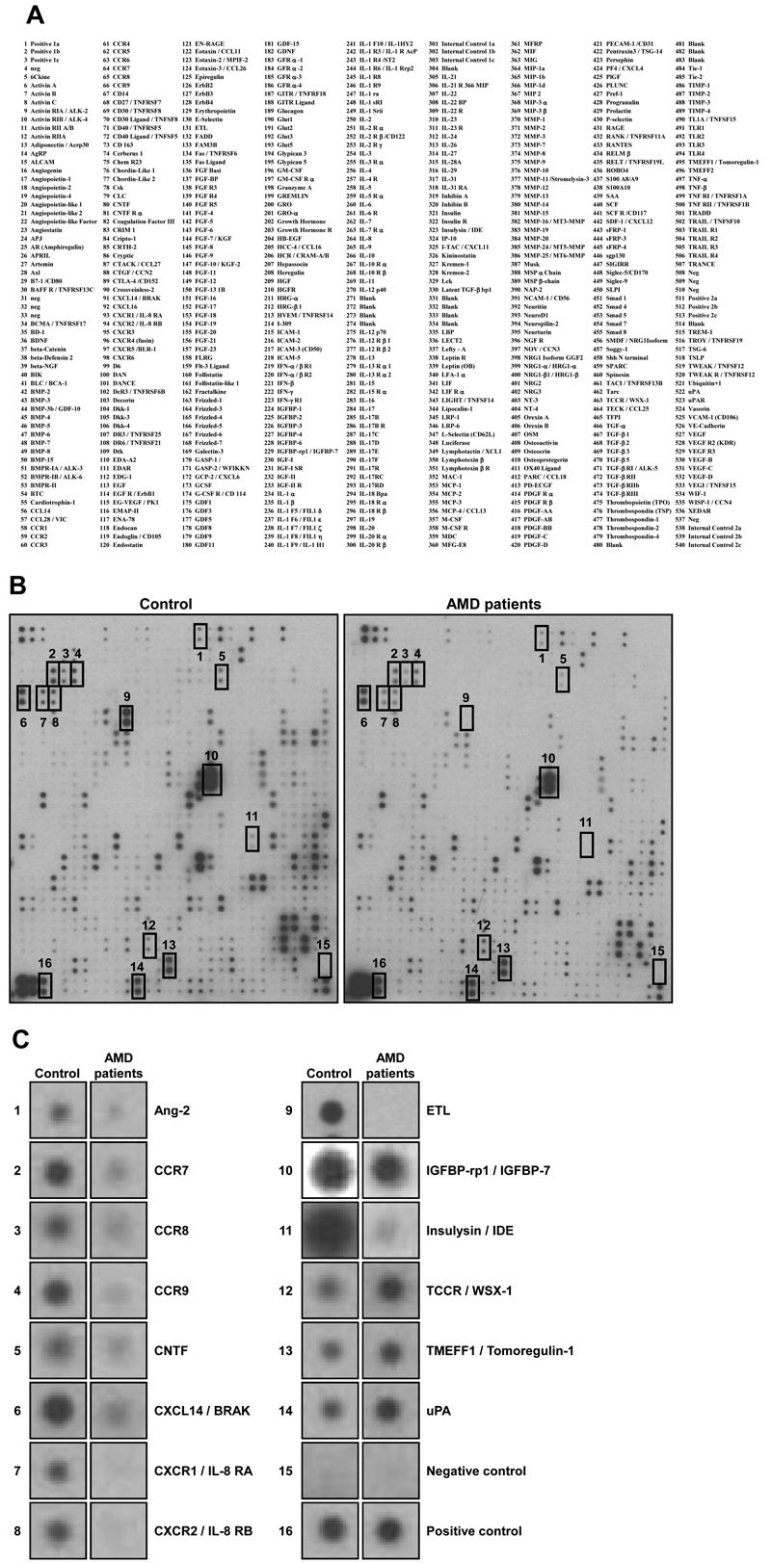
Expression levels of 507 proteins in aqueous humor of control or patients with age-related macular degeneration (AMD). **A**: The list of human antibodies. **B**: The mixed aqueous humor samples were assayed directly with the RayBio^®^ Biotin Label-based Human Antibody Array kit. **C**: The following proteins were changed in the aqueous humor of patients with AMD compared to control: Ang-2, CCR7, CCR8, CCR9, CNTF, CXCL14, CXCR1, CXCR2, ETL, IGFBP-7, and Insulysin (down-regulated); TCCR, TMEFF1, and uPA (upregulated). Negative control (15) and positive control (16) represent internal controls.

To identify novel secretory proteins for which the number changed in patients with AMD, we analyzed aqueous humor by using a membrane-based human antibody array kit. For convenience, all 30 samples were mixed and subjected to antibody-array analysis. Using an array designed to detect 507 proteins (Figure 1A), we found that the number of several proteins were changed in the aqueous humor of patients with AMD compared to the control (Figure 1B). Angiopoietin-2 (Ang-2), C-C motif chemokine receptor 7 (CCR7), CCR8, CCR9, ciliary neurotrophic factor (CNTF), C-X-C motif chemokine ligand 14 (CXCL14/BRAK), C-X-C motif chemokine receptor 1 (CXCR1/IL-8 RA), CXCR2 (IL-8 RB), EGF-TM7 latrophilin-related protein (ETL), insulin-like growth factor binding protein-related protein 1 (IGFBP-rp1/IGFBP-7), and insulysin (insulin-degrading enzyme/IDE) were decreased in the aqueous humor of patients with AMD. On the other hand, the number of TCCR/WSX-1, transmembrane protein with EGF-like and two follistatin-like domains 1 (TMEFF1/Tomoregulin-1), and u-plasminogen activator (uPA) were increased in patients with AMD (Figure 1C).

### Protein quantification

**Figure 2:**
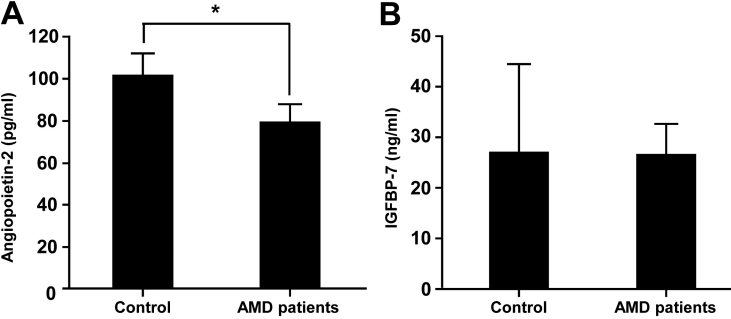
Enzyme-linked immunosorbent assay (ELISA) data of Ang-2 and IGFBP-7. The protein levels of Ang-2 (**A**) and IGFBP-7 (**B**) in aqueous humor samples were measured using the ELISA kit (* p=0.074).

To verify each sample, we next used quantitative analysis. Among the candidate proteins shown in Figure 1B, Ang-2, IGFBP-7, and uPA are available for ELISA analysis. As shown in Figure 2A, Ang-2 was decreased by about 20% in patients with AMD compared with the control group (p=0.074). IGFBP-7, however, did not appear to be different between the groups (Figure 2B), and the level of uPA was not in the detectable range (data not shown).

**Figure 3:**
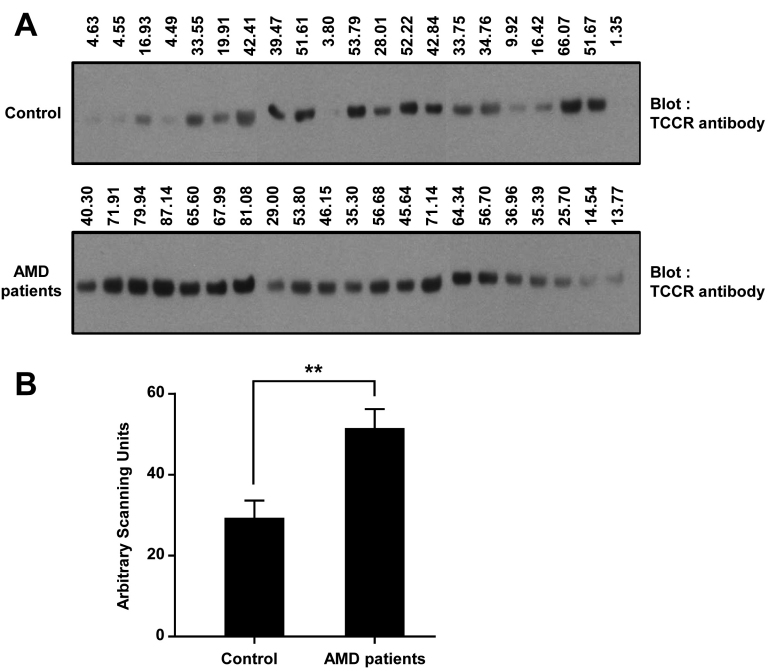
Western blot of T-cell cytokine receptor (TCCR). **A**: Twenty-one aqueous humor samples of the control and the age-related macular degeneration (AMD) patient groups were assessed with western blot using a specific antibody against TCCR. Upper numbers indicate the value by densitometry. **B**: The western blot data were quantified with a densitometer (** p=0.0013). The y-axis represents arbitrary scanning units.

We also verified the number of proteins by using western blot. Twenty-one samples in the control and AMD patient groups were used for western blot using a specific antibody against TCCR (Figure 3A). Densitometry data showed that TCCR was increased significantly in the aqueous humor of patients with AMD compared to the control group (p=0.0013) (Figure 3B).

### TCCR promotes angiogenesis of human umbilical vein endothelial cells in vitro

**Figure 4:**
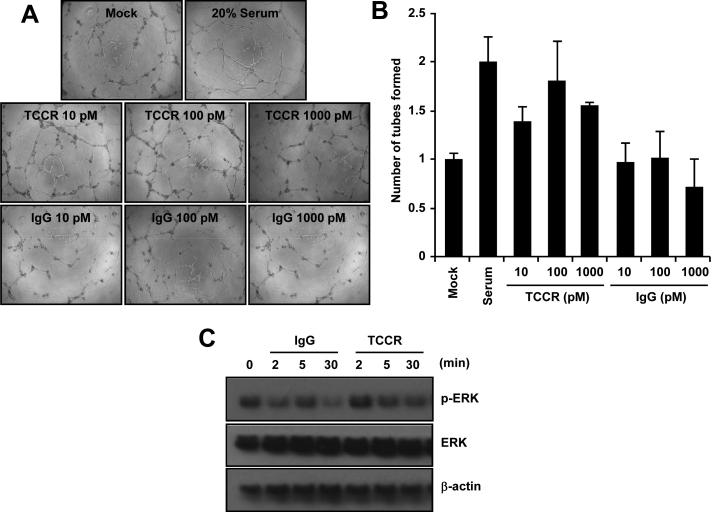
Effects of exogenous T-cell cytokine receptor (TCCR) on human umbilical vein endothelial cells (HUVECs). **A**: After HUVECs were treated with recombinant human TCCR/WSX-1/Fc chimera protein for 8 h, tube formation was detected with phase contrast microscopy. Twenty percent fetal bovine serum (FBS) was used as a control. **B**: The number of tubes was quantified by counting the total tubes per five fields in a blinded manner. **C**: western blot using antibody against ERK, phosphorylated extracellular signal-regulated kinase (ERK), and β-actin from HUVECs that were treated with 100 pM recombinant TCCR or control IgG for the indicated times.

To test whether TCCR could induce angiogenesis in endothelial cells, tube formation was measured using HUVECs in vitro. We found that exogenous human TCCR/WSX-1/Fc chimera protein promoted tube formation in HUVECs (Figure 4A,B). However, the control IgG did not show a similar outcome. ERK activation has been known to be closely associated with tube formation of HUVECs. As shown in Figure 4C, phosphorylation of ERK was increased transiently after treatment with the TCCR protein. Taken together, these data show that TCCR activates ERK and stimulates tube formation in endothelial cells.

## Discussion

In the present study, TCCR was significantly increased in the aqueous humor of patients with AMD. Moreover, treatment of endothelial cells with the extracellular domain of TCCR induced angiogenesis. There are two possible pathways by which TCCR could induce angiogenesis: First, TCCR acts as a neutralizer of IL-27 and thus inhibits the antiangiogenic effect of IL-27. IL-27 is known to bind the TCCR/gp130 receptor complex, activate the janus kinase/signal transducer and activator of transcription (JAK/STAT) pathway, and induce the expression of antiangiogenic molecules, CXCL9 and CXCL10. However, in the absence of gp130, IL-27 just binds to TCCR and cannot transduce its antigangiogenic signal to downstream. Second, soluble TCCR itself may act as a trigger for angiogenesis in this process.

TCCR (also known as WSX-1 and IL-27 Rα) is a 96-kDa type I cytokine receptor that functions as the ligand-binding component of the receptor for IL-27 and functions with the glycoprotein 130 (gp130) coreceptor to induce signal transduction in response to IL-27. Upon binding of IL-27 to its receptor complex TCCR/gp130, JAK1, STAT1, and STAT3 are activated [11,12]. Several years ago, Feng et al. [13] performed microarray experiments in primary human endothelial cells by treatment with IL-27. The study showed that IL-27 can induce the expression of CXCL9 and CXCL10 by 10- and nearly 60-fold in HUVECs. Both molecules are known to inhibit tumor growth and metastasis due to their antiangiogenic effects [14,15]. Therefore, the previous study clearly indicates that IL-27 acts as an inhibitor of angiogenesis. This may be of clinical importance, since in normal tissues only a small percentage of vascular endothelial cells are positive for CXCR3, which is a receptor for IL-27-induced CXCL10 [16]. The antiangiogenic effect of IL-27 is derived from macrophages and dendritic cells. The M1 phenotype of macrophages has antiangiogenic properties by producing IL-12 and interferon alpha (IFN-α)-related chemokines CXCL9, CXCL10, and CXCL11 [17,18]. In addition to macrophages, recent reports have shown that dendritic cells have direct and indirect effects on angiogenesis [19,20]. Certain chemokines, such as chemokine (C-C motif) ligand 2 (CCL2) and chemokine (C-X3-C motif) ligand 1 (CX3CL1), appear to be crucial for the accumulation of the subretinal microglia and macrophages observed in AMD and to participate in the development of retinal degeneration as well as in choroidal neovascularization [21]. This relates to the accumulation of macrophages in the subretinal space by CCL2 and CX3CL1. The prolonged presence of macrophages in the subretinal space is associated with photoreceptor degeneration [22,23] and the development of CNV in animal models [24,25], which have been reported to involve possible induction of angiogenesis. Taken together, increased TCCR in patients with AMD may reduce the expression of CXCL9 and CXCL10 by neutralizing the effect of IL-27 with the outcome being an angiogenic effect.

We also suggest the possibility that soluble TCCR may serve as a ligand for angiogenesis. Several receptors have dual roles for ligands as well as for the receptor itself. For example, ephrins can activate forward signaling to Eph receptors and reverse signaling through their cytoplasmic domain [26]. In our study, we found that TCCR existed in the aqueous humor is elevated in patients with AMD and the exogenous extracellular domain of TCCR itself activates a signaling pathway and induces angiogenesis of endothelial cells. Although we could not determine whether the level of the whole TCCR protein changed and how TCCR was processed to its cleaved form in patients with AMD, our study suggests a novel function of TCCR in addition to its receptor function against IL-27.

Although the precise mechanism of action of TCCR in angiogenesis must be investigated more, inhibition of TCCR signaling may be a possible strategy for application in patients with AMD. Some examples are the neutralizing antibodies against soluble TCCR, the small molecules inhibiting TCCR cleavage, and the chemicals blocking the downstream signaling induced by soluble TCCR-receptor coupling. If the molecules with these inhibitory properties could be found and are available, combinatorial therapeutic application of these molecules with established antiangiogenic agents might be useful in AMD therapy.

To conclude, we propose that the soluble form of TCCR/WSX-1 induces angiogenesis in the aqueous humor and therefore may be a potential therapeutic target in AMD.
